# A comparison of cancer burden and research spending reveals discrepancies in the distribution of research funding

**DOI:** 10.1186/1471-2458-12-526

**Published:** 2012-07-17

**Authors:** Ashley JR Carter, Cecine N Nguyen

**Affiliations:** 1 Biological Sciences Department, California State University Long Beach, 1250 Bellflower Blvd, Long Beach, CA, 90840, USA

**Keywords:** Cancer, Research funding, Years of life lost, YLL, Disability adjusted life years, DALY, Burden, Economic cost

## Abstract

**Background:**

Ideally, the distribution of research funding for different types of cancer should be equitable with respect to the societal burden each type of cancer imposes. These burdens can be estimated in a variety of ways; “Years of Life Lost” (YLL) measures the severity of death in regard to the age it occurs, "Disability-Adjusted Life-Years" (DALY) estimates the effects of non-lethal disabilities incurred by disease and economic metrics focus on the losses to tax revenue, productivity or direct medical expenses. We compared research funding from the National Cancer Institute (NCI) to a variety of burden metrics for the most common types of cancer to identify mismatches between spending and societal burden.

**Methods:**

Research funding levels were obtained from the NCI website and information for societal health and economic burdens were collected from government databases and published reports. We calculated the funding levels per unit burden for a wide range of different cancers and burden metrics and compared these values to identify discrepancies.

**Results:**

Our analysis reveals a considerable mismatch between funding levels and burden. Some cancers are funded at levels far higher than their relative burden suggests (breast cancer, prostate cancer, and leukemia) while other cancers appear underfunded (bladder, esophageal, liver, oral, pancreatic, stomach, and uterine cancers).

**Conclusions:**

These discrepancies indicate that an improved method of health care research funding allocation should be investigated to better match funding levels to societal burden.

## Background

Cancer is the second leading cause of death in the United States, accounting for over 550,000 deaths in 2010 [1], and the fear of cancer is an ever-present specter in society. Consequently, the government funds a considerable amount of medical research designed to understand the processes involved in cancer progression with the goal of improved treatments. Given that the total amount of money devoted to improving public health is limited, improvements in the allocation of funding can benefit society at no additional cost. If the primary goal of cancer research is to reduce the deleterious effects cancer imposes on society and victims, two issues must be addressed to enact a cancer research policy that best achieves this goal. The issues are (1) the choice of one or more appropriate metrics of cancer burden and (2) the optimization of research spending with respect to these burden metrics [2].

The most straightforward burden measurement is to count raw mortality, the number of deaths caused by cancer. Although this metric is the easiest to calculate (death records are among the most reliable health records) and the easiest to interpret, this metric alone is incomplete. For example, one can argue that a disease that killed 100,000 children demands more attention than a disease that killed 100,000 people exclusively aged 80 years and older. Similarly, if two diseases are identical in all aspects except that one is ten times more expensive due to inefficient treatment, more attention should be given to improving the treatment of the more costly disease.

Weighting the severity of death by the age a person is killed is calculated by the metric broadly termed "Years of Life Lost (YLL; sometimes also referred to as "Years of Potential Life Lost", YPLL or "Potential Years of Life Lost", PYLL). A number of variations of the YLL statistic exist [3], including ones that cap life expectancy tables at arbitrary values (essentially assigning no burden to death at older ages). We believe that deaths to individuals age 65 or 70 are a burden on society so we consider YLL values calculated from uncapped life expectancies. YLL is calculated by subtracting the age at death from the life expectancy based on the age bracket of the deceased. Conceptually, a 20 year-old male dying in 2010 with a projected life expectancy of 72.4 years would have lost approximately 52.4 years due to cancer. Alternatively, a 50 year-old male dying in 2010 with a projected life expectancy of 67.55 years would have lost only 17.55 years of life [4]. Actual YLL calculations are more complex and based on detailed life tables for individuals at certain ages. Since older individuals have already overcome some of their lifetime risk, their remaining years of life is higher than merely subtracting age at death from their original life expectancy at birth. The YLL for an individual therefore never declines to zero, but does decline as a person ages to reflect the amount of additional time they would have been expected to live.

In addition to YLL, many other health-based burden metrics have been proposed (e.g., disability-adjusted life years, DALY; Quality-adjusted life years, QALY; years lost to disability, YLD). These values represent lost quality of life due to non-lethal disabilities by translating these effects into years of life lost equivalents. For example, surviving a condition but incurring severely reduced mobility for the remaining years may result in someone living 30 additional years being considered to live 15 additional years instead due to the reduced quality of their life during those years. These metrics can be useful, but are somewhat subjective [5] and have a number of technical and theoretical weaknesses [6]. We favor the YLL metric because calculation is relatively straightforward; it weighs death at an early age higher than death later in life, and is relatively free of arbitrary judgment decisions. Actions that reduce the YLL will also tend to reduce DALY and QALY values as well by virtue of curing the disorders that cause the reduced quality of life and increased disability measured by those statistics. The use of DALYs is widespread however and previous studies have shown that funding decisions may be driven more strongly by DALY values than by mortality or YLL values [2,7] therefore we included an analysis of DALYs in this study.

Additional metrics examining cancer burden attempt to capture the economic costs. The YLL metric can be adjusted to include years of working age lost and provides the economic cost to society based on lost tax revenue and productivity (due to shortened careers and time taken off for treatment). The direct financial cost incurred by medical care for individuals undergoing treatment can also be estimated. The first of these metrics tends to weigh younger individuals higher, but only after they achieve working age. This means that the very young are assigned lower values, contrary to what many members of society may believe. Estimating the direct costs of medical care is complex as these values may vary widely by region, personal income, and individual insurance status.

Once a metric has been chosen, improved funding allocation can be achieved by distributing resources more equitably according to this metric; according to this logic, cancers with twice the burden should receive twice the funding. Arguments for inequity in spending relative to burden are equivalent to arguments for altering the burden metric used. For example, an argument that breast cancer deserves more funding than warranted (as defined by one of the burden metrics above) may be made due to the deep psychological importance and sense of identity women attach to their breasts. This argument requires that psychological factors should be added to the burden metric and shows the complexity involved in considering the estimation of burden. Careful attention to the metric used is essential because the relative burdens incurred by different types of cancer are likely to differ when using different metrics, sometimes strikingly so [8]. Because various metrics may be favored for different purposes, research spending should ideally be compared to a number of different metrics to allow a full consideration of cancer research policy to proceed.

A search of PubMed revealed a modest number of published studies comparing research funding to societal burden. Studies examining funding for broad categories of disease include: Australian government funding and grants awarded for a wide range of causes of death [9] and specific cancers [10,11], Spanish government funding for a wide range of causes of death [12], US research funding for a number of disorders by the National Center for Complementary and Alternative Medicine [5], all US public and private funding for a wide range of disorders [13] and UK funding for cancer, heart disease, stroke and dementia [14]. Only some of these studies focused on individual cancer types and compared research effort to individual and societal burden [8,10,11,15]. The discrepancies identified in these analyses differ from those we identify and discuss below.

Our study considers several metrics of burden in the US based on recent data and presents a series of comparisons to the National Cancer Institute (NCI) research funding levels. Several cancer types seem to be funded at levels out of alignment with their respective societal burdens. This disparity indicates an opportunity for improving the allocation of cancer research resources. We examine a number of different comparisons; this approach is beneficial because it fosters a more comprehensive understanding of the overall issue.

## Methods

The SEER Cancer Statistics Review 1975–2007 is published by the National Cancer Institute and makes estimations of national statistics based on direct information from 26% of the US population [16]. That document was used to obtain the values of incidence, mortality and YLL for different cancers (their Table 1.1 and Figure 1.19). Our own calculations (using raw data, not reported) and those estimated in [17] are in very close agreement with the SEER values (R^2^ > 0.98 for both comparisons). The "Average Years of Life lost" (AYLL) statistic is calculated by dividing the YLL value by the number of deaths for each cancer. The incidence and mortality values are estimates for 2010 while the YLL values are estimates for 2007. Rather than match the years for these values exactly, we elected to use the most recent values available.

**Table 1:** Raw values used for analyses

		**Incidence**	**Mort.**	**US Gov't** **YLL (thousands)**	**WHO** **DALY (thousands)**	**Medicare Spend$ (millions)**	**National spend $ (billions)**	**Lost** **prod. (billions)**	**NCI Funding (millions)**	**AYLL**	**YLLPI**
Bladder	Blad	70530	14680	154.4	128.7	1023	3.466	1.977	22.6	10.52	2.19
Brain, ONS	Br/cns	22020	13140	290.8		293	3.715	5.851	193.1	22.13	13.21
Breast	Breast	209060	40230	761.3	612.5	1375	13.886	10.879	631.2	18.92	3.64
Cervix	Cerv	12200	4210	104.7	114.1	73	1.425	1.808	76.5	24.87	8.58
Colon/rectum	Co/rec	142570	51370	764.6	542.1	3101	12.155	12.802	270.4	14.88	5.36
Esophogus	Esoph	16640	14500	214.2	122	386	1.071	---	30.5	14.77	12.87
Hogkin lymph.	H lym	8490	1320	29.8	288.7**	1350*	10.168*	0.829	14.6	22.58	3.51
Kidney	Kidney	58240	13040	195.7	---	685	3.058	3.633	90.0	15.01	3.36
Leukemia	leuk	43050	21840	355.2	210.5	695	4.507	5.880	295.8	16.26	8.25
Liver	Liver	24120	18910	292.2	137.8	278	---	4.638	72.6	15.45	12.11
Lung	Lung	222520	157300	2369	1247.6	4238	10.315	38.953	281.9	15.06	10.65
Myeloma	Myel	20180	10650	148.1	**	---	---	---	48.5	13.91	7.34
NH Lymphoma	NHL	65540	20210	292.4	**	*	*	5.755	122.4	14.47	4.46
Oral,pharnyx	Oral	36540	7880	138	85.4	---	---	---	13.9	17.51	3.78
Ovary	Ovary	21880	13850	248.8	145	507	4.379	2.945	112.3	17.96	11.37
Pancreas	Panc	43140	36800	498.4	237.7	771	1.884	7.058	97.1	13.54	11.55
Prostate	Pros	217730	32050	267.4	225	2294	9.862	3.538	300.5	8.34	1.23
Melanoma (skin)	Melo	68130	8700	150.7	120.6	181	1.906	3.298	102.3	17.32	2.21
Stomach	Stom	21000	10570	176	106.9	624	1.550	3.454	14.5	16.65	8.38
Testes	Test	8480	350	11.8	---	---	---	0.472	6.3	33.71	1.39
Uterus	Uter	43470	7950	122.8	75.5	340	2.330	1.101	14.2	15.45	2.82

Incidence, mortality, YLL, three measures of economic cost (Medicare payments, overall medical spending, and lost productivity), research funding by the NCI and calculated values for AYLL and YLLPI using data from the table for the 21 cancers for which we had sufficient data. The symbol --- indicates that data for those values were not available, * indicates that for Medicare and National expenditure cost estimations the categories "Hodgkin's lymphoma" and "non-Hodgkin's lymphoma" were combined in those reports, ** indicates that for for World Health Organization (WHO) DALY estimates the categories "Hodgkin's lymphoma", "non-Hodgkin's lymphoma" and "myeloma" were combined in those reports.

**Figure 1:**
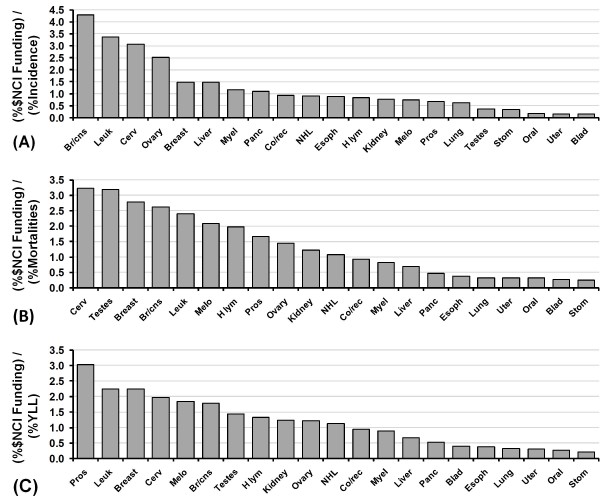
**NCI research funding relative to societal health cost.** NCI research funding relative to societal health cost. Values are the amount of NCI funding for each cancer expressed as a percentage of overall funding divided by the (**a**) percentage of incidences, (**b**) percentage of mortalities or (**c**) value of YLL as a percentage of the overall value for all 21 cancers.

We used information available online from the World Health Organization [18] to obtain the estimates of United States DALY values for most of the cancers used in our analysis. Those records omitted liver cancer and combined non-Hodgkin's and Hodgkin's lymphoma with myeloma into a single category.

We examine an additional value which we term the "Years of Life Lost Per Incidence" (YLLPI), which is calculated by dividing the YLL by the number of new cases. Whereas the AYLL estimates the years of life lost to individuals that die, the YLLPI estimates the expected years of life lost to an individual who is diagnosed with cancer, including their chances of successful treatment with no years of life lost. The YLLPI therefore includes information about treatment efficiency (cancers with high cure rates result in lower YYLPI values) and the potential years of life lost (age at affliction enters the calculation by use of the YLL). We believe this metric best represents the potential benefit accrued from research by combining the potential for improved outcome (reducing a high mortality rate) with the value of improved outcome (favoring cures for the young over cures for the elderly). To our knowledge this statistic has not been widely considered.

Values for research funding categorized by cancer type were obtained from the NCI [19]. The categories for cancer type are not identical in the NCI and SEER sources described in the preceding paragraph. Hence, some funding categories were combined in order to match the SEER categories as follow: (i) brain, nervous system and central nervous system categories were combined and assigned to "brain, ONS"; (ii) kidney cancer and kidney disease were combined and assigned to "kidney"; (iii) leukemia and childhood leukemia were combined and assigned to "leukemia", (iv) buccal cavity and pharynx were combined and assigned to "oral, pharyngeal".

Medical care for cancer patients represents a major part of the economy and estimates of the direct cost of cancer care can be made in different ways. Yarboff et al. [20] used diagnosis and Medicare payment data from 2004 to estimate the 5-year cost of care for individuals and calculated this for most of the cancers we consider in this study (Their analysis omits estimates for myeloma, oral/pharyngeal and testes cancer and combines Hodgkin's and non-Hodgkin's lymphomas). Their raw numbers represent a subset of all health care spending on these cancers and can't be used in direct calculations with the values obtained from our other sources. For the purpose of calculating the treatment cost of each cancer, we do not expect relative costs to vary widely when payment is made by other sources. A second set of data for costs was obtained directly from the NCI website [21]. Their figure LCO1 provides the estimated national expenditures for cancer care in 2006 for most of the cancers we consider in this study (Their figure omits estimates for myeloma, oral/pharyngeal, liver and testes cancer and combines Hodgkin's and non-Hodgkin's lymphomas).

A measure of the economic burden incurred by lost productivity due to cancer comes from estimating the "present value of lifetime earnings" (PVLE) of all individuals that die from cancer in a given year [22]. This calculation weighs working-age individuals higher than the elderly (their remaining earnings are higher), weighs males higher than females (males have higher average incomes), and weighs racial and ethnic groups differently (groups with lower average incomes are weighed lower while those with higher average incomes are weighed higher). Placing a dollar value on a life based on economic calculations is routinely performed by governmental agencies such as the Environmental Protection Agency ($7.4 M in 2006 dollars; [23]) and the Federal Highway Administration ($2.6 M in 1994 dollars; [24]). We obtained PVLE values for individuals that die from various cancer types from Table 2 of [22]; that source included values for most, but not all, cancers we consider in this study (PVLE values for esophageal, myeloma and oral/pharyngeal cancers were not reported).

A number of the calculations performed and values plotted in the next section compare the percentage of overall funding, cost or mortality for each cancer type to one another. Unless otherwise noted, the overall funding, cost or mortality (including YLL, etc.) value is calculated from the sum of those listed and does not include additional ones not plotted. For example, in comparisons of %YLL and %Medicare costs for various cancers, the overall YLL used for the %YLL calculations does not include myeloma, testes cancer and oral cancer as Medicare estimates were not available in our data source. This discrepancy can result in slight differences in %YLL values for each cancer in different comparisons, depending on which data was available. In each comparison, we identify which cancers are included and excluded in our analysis.

Due to its high incidence, prevalence and mortality rates, lung cancer represents an outlier in many statistical and social considerations of cancer prevention and treatment funding. In comparative terms, lung cancer causes more cases of cancer than any other type and three times as many deaths as the next two most lethal types of cancers (colo-rectal and breast). Meanwhile, lung cancer receives less funding than breast, prostate and leukemia and roughly the same funding as colorectal cancer, which kills less than a third as many individuals. In absolute terms, lung cancer accounts for 32% of cancer deaths while receiving 10% of cancer research funding. Due to the extreme nature of incidence, prevalence and mortality values for lung cancer, some of the analyses presented in this paper omit lung cancer because its inclusion would obscure the recognition of patterns identified in the rest of the data.

## Results

Raw data for the top 21 cancers are presented in Table 1. This table provides data on the different variables used for comparison in the study. Figure 1 shows the percentage of overall NCI research funding on these cancers relative to the overall percentage of incidences, mortalities and YLL expressed as a ratio - a general indication of overfunding (ratio > 1) or underfunding (ratio < 1). The ratios indicate large differences in funding per incident, mortality and YLL for different cancer types. The cancers that appear to be the most overfunded depend on the metric chosen, but similar discrepancies occur multiple times. Considering incidence, overfunded cancers appear to be brain/CNS, cervical, leukemia, and ovarian. Considering mortalities, overfunded cancers appear to be brain/CNS, breast, cervical, leukemia, and testes. Considering YLL, overfunded cancers appear to be brain/CNS, breast, cervical, leukemia, melanoma, and prostate. The cancers that appear to be the most consistently underfunded across the different metrics are bladder, esophageal, lung, oral, stomach, and uterine cancers.

**Figure 2:**
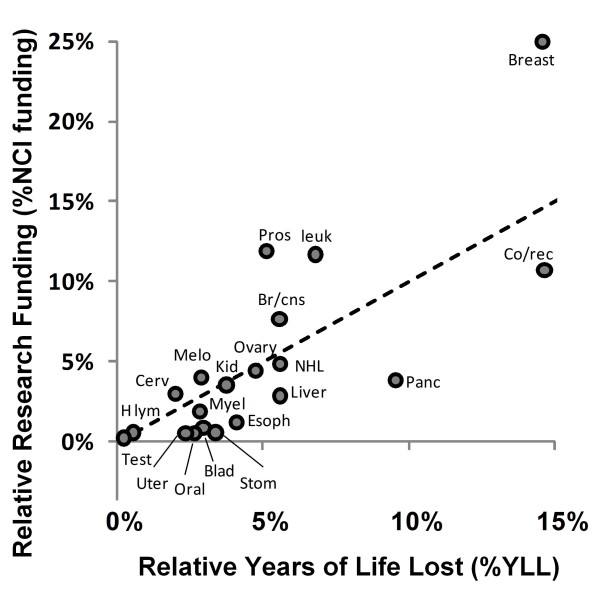
**NCI research funding relative to societal health cost as measured by YLL.** Dashed line indicates funding if resources are equitably shared according to burden imposed by YLL, cancers above the line receive more funding relative to YLL than expected whereas cancers below the line receive less. Due to its extremely large %YLL (approx. 30%) and relatively low funding (approx. 10%), lung cancer is excluded from this plot and was not used in these calculations.

Figure 2 shows a plot of %NCI funding vs %YLL; data points deviating from the 45 degree line of equitable funding indicate over or under funding (according to a goal of minimizing YLL) in absolute terms. Three cancer types have extremely positive deviations indicating overfunding (breast, leukemia, and prostate) and one other has a moderately positive deviation (brain/CNS). The negative deviations indicate that pancreatic cancer appears the most underfunded, with bladder, colorectal, esophageal, liver, oral, stomach, uterine cancers moderately underfunded.

**Figure 3:**
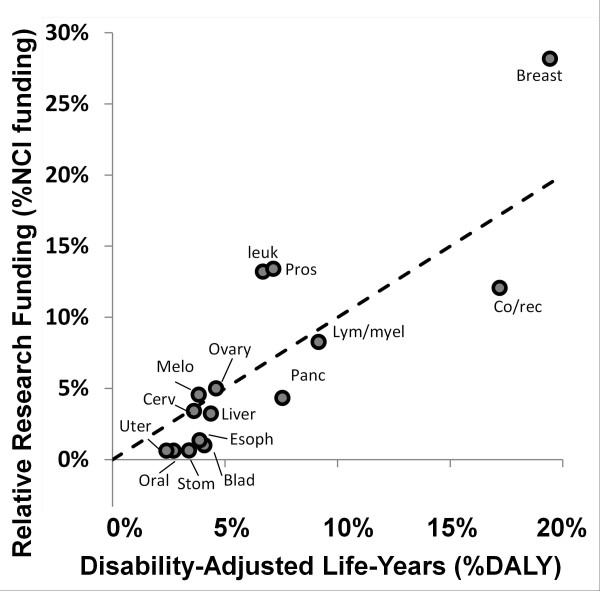
**NCI research funding relative to societal health cost as measured by DALYs.** Dashed line indicates funding if resources are equitably shared according to burden imposed by DALY, cancers above the line receive more funding relative to DALY than expected whereas cancers below the line receive less. Due to its extremely large %DALY (approx. 30%) and relatively low funding (approx. 10%), lung cancer is excluded from this plot and was not used in these calculations.

Figure 3 shows a plot of %NCI funding vs %DALY; data points deviating from the 45 degree line of equitable funding indicate over or under funding (according to a goal of minimizing YLL) in absolute terms. Similar to figure 2, three cancer types have extremely positive deviations indicating overfunding (breast, leukemia, and prostate). The negative deviations indicate that bladder, colorectal, esophageal, oral, pancreatic stomach and uterine cancers appear underfunded.

**Figure 4:**
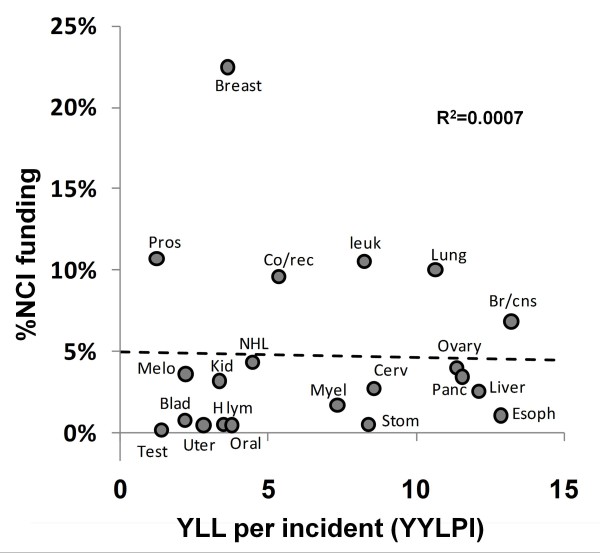
**NCI research funding relative to YLLPI.** Dashed line indicates linear regression and the R^2^ of 0.0007 indicates that NCI research funding is not associated with YYLPI. Removal of the extreme value for breast cancer from this plot does result in a slight non-significant positive relationship (R^2^ = 0.0223).

Figure 4 indicates that NCI research funding at present is not associated with YYLPI. Research efforts are not directed at cancers for which the YLLPI statistic suggests that research is more likely to meet with beneficial results.

**Figure 5:**
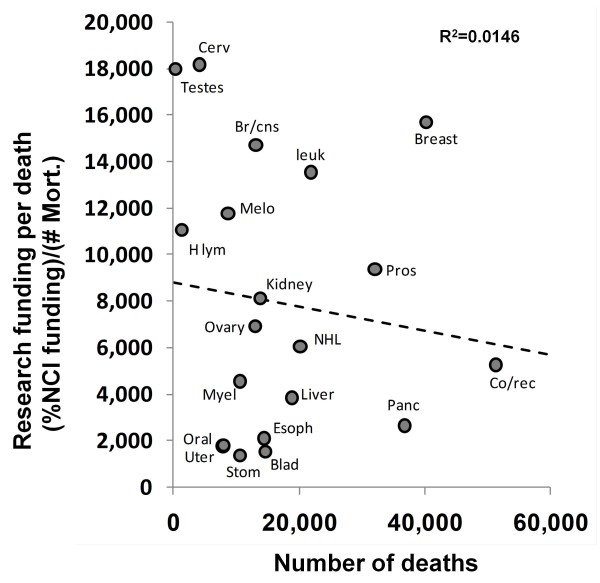
**NCI research funding relative to total number of deaths.** Dashed line indicates linear regression and the R^2^ of 0.0146 indicates virtually no relationship between the number of deaths due to each cancer and the funding per death. Due to its extremely large number of deaths (approx. 160,000) and relatively low funding (approx. $1800 per death), lung cancer is excluded from this plot. Removal of the extreme value for testes cancer from this plot reverses the relationship (positive slope, R^2^ = 0.008), but it remains non-significant.

Figure 5 compares the funding per mortality to the total number of mortalities. A situation where the quantity of deaths influences funding may cause a mismatch in funding prioritization. Due to the absence of a pattern in this data (negative slope, R^2^ = 0.0146), high profile cancers that cause more deaths do not appear to receive more funding than expected based on the number of overall deaths.

**Figure 6:**
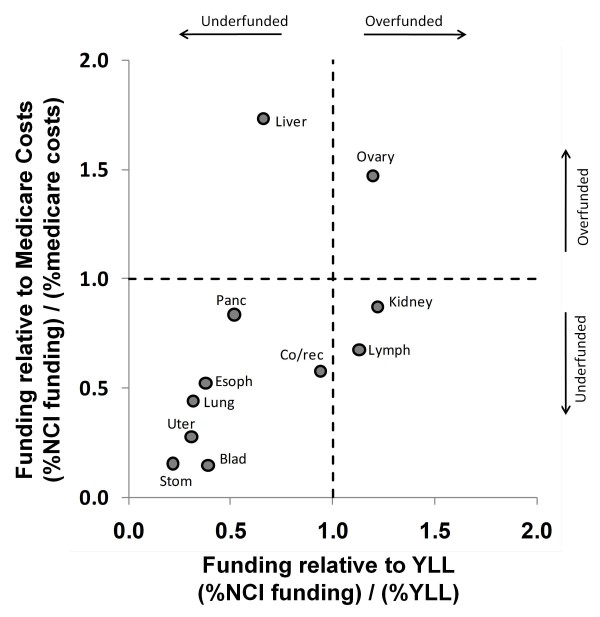
**NCI research funding relative to Medicare payment costs[**[20]**]and YLL.** Dashed lines indicate borders between regions that are underfunded (%funding < % cost or %YLL) and overfunded (%funding > % cost or %YLL). Several cancers have funding ratios exceeding 2.0 for one axis or the other and are therefore considerably overfunded according to the criteria corresponding to that axis. To focus on underfunded cancers, those cancers (breast, leukemia, melanoma, prostate, and brain/cns) are not depicted. Note also that economic data for myeloma, oral cancer and testes cancer was not available and the lymphoma data represents both Hodgkin's and non-Hodgkin's lymphomas combined.

**Figure 7:**
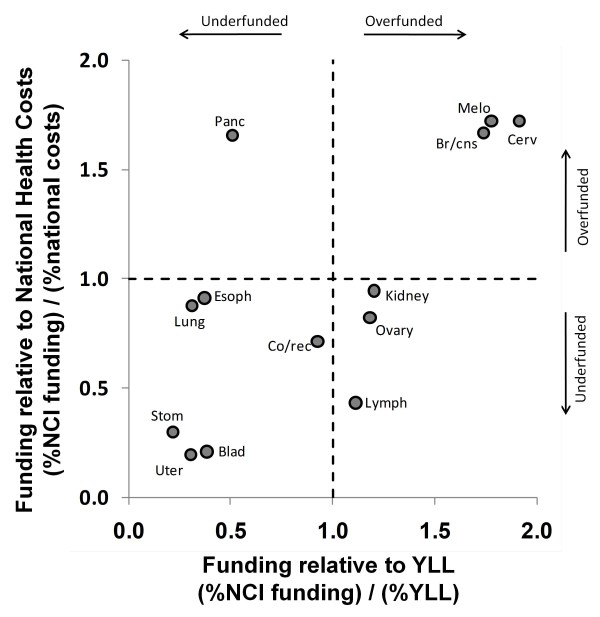
**NCI research funding relative to estimated Total National medical costs[**[21]**]and YLL.** Dashed lines indicate borders between regions that are underfunded (%funding < % cost or %YLL) and overfunded (%funding > % cost or %YLL). Several cancers have funding ratios exceeding 2.0 for one axis or the other and are therefore considerably overfunded according to the criteria corresponding to that axis. To focus on underfunded cancers, those cancers (breast, leukemia and prostate) are not depicted. Note also that economic data for liver cancer, myeloma, oral cancer and testes cancer was not available and the lymphoma data represents both Hodgkin's and non-Hodgkin's lymphomas combined.

**Figure 8:**
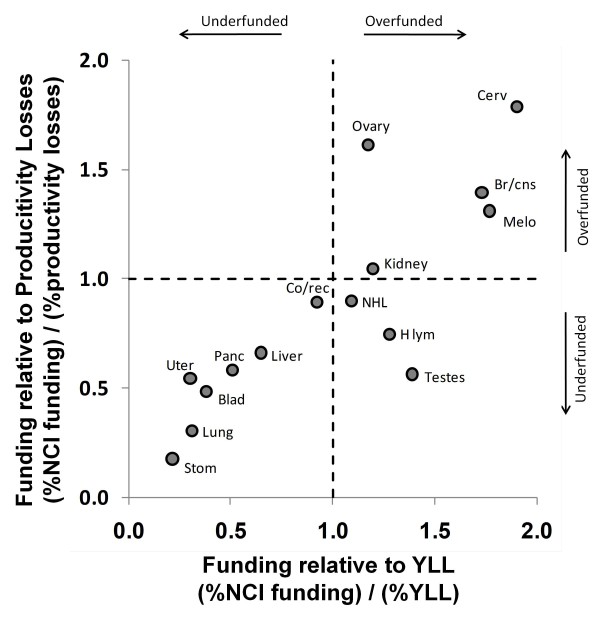
**NCI research funding relative to economic costs arising from lost productivity and earnings[**[22]**]and YLL.** Dashed lines indicate borders between regions that are underfunded (%funding < % cost or %YLL) and overfunded (%funding > % cost or %YLL). Several cancers have funding ratios exceeding 2.0 for one axis or the other and are therefore considerably overfunded according to the criteria corresponding to that axis. To focus on underfunded cancers, those cancers (breast, leukemia and prostate) are not depicted. Note also that economic data for esophageal cancer, myeloma and oral cancer was not available.

Figures 6, 7 and 8 show the relationship between funding relative to economic costs and funding relative to YLL. Figure 6 calculates relative economic costs based on Medicare payments using data in [20]. Figure 7 calculates relative economic costs based on overall national spending on medical care for these conditions using data in [21]. Figure 8 calculates relative economic costs based on the total loss of productivity incurred by treatment and deaths from these cancers (mainly from reductions in lifetime earnings by decedents) using data in [22]. Cancers that appear in the lower left of these plots (bladder, stomach, and uterine) are underfunded according to both the YLL and economic metrics.

## Discussion

The National Institutes of Health (NIH) has recommended that research effort be compared to the societal burden of that disease [2]. Our analysis indicates that the amount of NIH funding for research on a disease is associated with the burden of the disease, but discrepancies exist. Different measures of the burden of disease yielded different conclusions about the degree to which some cancer types were over or underfunded; these general conclusions are in agreement with the study by Gross et al. [2] from 1999 but also indicate that more than a decade later these discrepancies persist.

For example, Figure 1 shows that stomach cancer receives the least amount of funding relative to its burden; stomach cancer receives less than 10% the amount of funding, per death and YLL, of breast cancer. Similarly, research funding to treat uterine cancer, per death and YLL, is 10% the funding given to treat testes cancer.

Figures 1, 2, 3, 4 and 6, 7, 8 allow us to identify the following cancers as funded at levels far below others which incur similar costs to society: bladder, oral, uterine, and stomach. A degree of underfunding also applies to esophageal, liver, and pancreatic cancers. Given their high YYLPI values, they represent promising areas for potential gain compared to cancers that have already achieved low mortality rates. Conversely, research on breast cancer, leukemia, and prostate cancer appear to be higher than justified relative to their burden on society as measured by YLL and economic costs. In summary, based on our data, we recommend that funding resources be directed away from breast cancer, prostate cancer, and leukemia toward bladder, esophageal, liver, oral, pancreatic, uterine, and stomach cancers.

To illustrate the type of redistribution we suggest, consider Figure 2. Reducing the funding for leukemia research to an equitable level with regard to YLL (from 11.70% of funding to 6.81%) would free more than enough resources to raise bladder and uterine cancer funding to equitable levels with regard to YLL (0.9% to 2.96% and 0.56% to 2.35% respectively), therefore creating a better overall distribution of funding relative to the societal burden measured by YLL. As a second example, plots 2 and 3 show that reducing breast cancer funding alone to an equitable level would provide more funding that could be used to raise several of these underfunded cancers up to parity.

The use of DALYs has received much attention in the prioritization of funding and studies have indicated that funding is more tightly correlated with DALY values than with incidence, mortality or YLL values [5]. Figure 3 illustrates a similar pattern of funding distribution relative to the different cancer types as seen in Figure 2 with the exception that liver cancer appears more equitably funded when using DALY values instead of YLL values. Use of DALY values is controversial for two main reasons [25,26]. First, the weights used are determined by indirect methods rather than methods measuring impact of the disabilities directly. Second, the estimation and true cost of disability may vary in different regions and within different subpopulations. Wealthier regions and populations are better equipped to accurately estimate the factors that go into the DALY weighting values and may weight certain behavioral factors differently than less wealthy groups. The conclusions of the analyses using both the YLL and DALY values are similar therefore the controversial aspects of DALY valuations do not pose a problem for our overall recommendations.

We believe that considering the "Years of Life Lost Per Incidence" (YLLPI) is useful for making research decisions. Research to treat cancer is often concerned with improving the outcome of people afflicted. With the exception for special circumstances like the HPV vaccine (which reduces the risk of cervical cancer), most NCI funded research is aimed at reducing mortality instead of incidence. If the number of cases is mainly due to factors like behavior and genetics that are hard to influence, plots of research funding vs. YLLPI (Figure 4) may provide the clearest identification of situations where increased funding has the best potential to improve treatment. This comparison does not include the number of individuals that may be affected by improved treatment, but does estimate the "room for improvement" per treatment. Cancers that kill younger individuals and have high mortality rates tend to have a higher YLLPI. Marginal improvement in treatment for these conditions may be a more realistic goal and provide the same or greater benefit than improvement in conditions with low YLLPI because they already have low mortality rates or tend to affect the elderly.

The economic costs of cancer are also part of the overall consideration of research funding allocation. In Figures 6, 7, 8 we compare funding relative to several economic cost metrics and funding relative to YLL for each cancer. The first two of these metrics (Medicare payments and estimated medical care) measure direct medical care costs. The third metric (lost productivity) attempts to make a more comprehensive estimate of all costs associated with cancer. Cancers in the lower left of these plots appear to be underfunded according to both the economic and YLL metrics. In addition to arguments about reducing suffering, purely economic arguments would favor increasing funding for cancers in the lower left at the expense of decreased funding for cancers not in these regions. This analysis indicates that the most efficient changes in research allocation, from a purely financial perspective, involve reducing funding for leukemia, melanoma, breast, and prostate cancers while increasing funding for bladder, stomach, and uterine cancers.

Comparison of our results with previous similar studies reveals some similar conclusions and some differences.

Incidence and mortality values were used in a Canadian study by Branton [15] and in a strategic analysis document published by the UK National Cancer Research Institute [27]. For the Canadian data, colorectal and lung cancers were identified as underfunded relative to other cancers. The UK analysis identified leukemia, ovarian and cervical cancers as overfunded whereas lung, pancreatic, stomach, esophageal and bladder cancers were identified as underfunded. These results generally agree with ours which may reflect similar approaches to research science in the US, UK and Canada.

Years of Life Lost values were used in studies of Australian data. An analysis of overall Australian funding per YLL showed that breast, cervical, leukemia, melanoma and prostate cancers were funded at far higher levels than provided for bladder, brain, gall bladder, lung/mesothelioma, kidney, lymphoma and pancreatic cancers [10]. Interestingly a separate analysis of Western Australia showed similar results with the exception that a large number of studies of mesothelioma were funded [11], likely due to the history of asbestos mining in the region.

Burnet et al. [8] performed a study of cancer funding and societal burden as measured by YLL statistics Using data from the UK (life expectancy data from 1990 life tables and funding data from a 2002) to identify apparent cases of funding discrepancy.

In their AYLL ("Average Years of Life Lost" which is YLL per death) analysis they describe a "Cinderella" region with high AYLL and low funding to identify cancers they believe deserve additional funds. However, focusing on AYLL ignores the total number of cases and the per incidence mortality rate in the burden completely. For example, using their method, a cancer that kills a single child each year would be placed within their "Cinderella" region and be recommended for more funding. This approach would identify testes cancer as greatly underfunded even though it has the lowest rate of incidence and the lowest rate of mortality per incidence of the cancers we examined, resulting in only 350 deaths in 2010. By focusing on AYLL alone, two of the four cancer types they identify as underfunded (cervical cancer and melanoma) are misidentified and appear overfunded when using YLL as a metric. Furthermore, the six types of cancer we identify as underfunded do not appear in their "Cinderella" region of underfunded cancer types.

**Figure 9:**
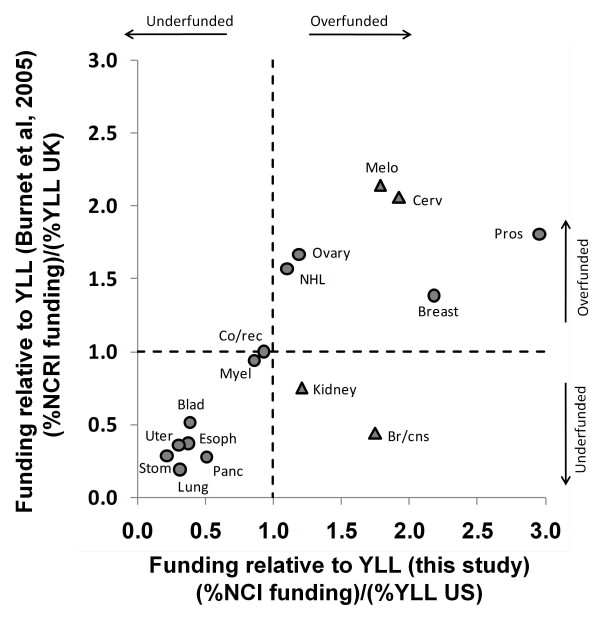
**Comparison of research funding relative to YLL as reported for the UK[**[8]**]and in this study for the US.** Dashed lines indicate borders between regions that are underfunded (%funding < % cost or %YLL) and overfunded (%funding > % cost or %YLL). Cancers identified with triangles are those named in the text (and abstract) of Burnet et al. [8] as lying in a "Cinderella" region indicative of underfunding. Several cancers are identified as underfunded in both studies, but only two of the "Cinderella" region cancers appear underfunded according to the UK data and none are underfunded according to our US data. Data for for Hodgkin's lymphoma, liver cancer, oral cancer and testes cancer are not plotted as they were not included in the UK data [8]. Due to its extremely large ratio in the UK study (approx. 5.83) leukemia is excluded from this plot although the values were used in the calculation of the relative funding and YLL values for the remaining cancer types.

In their second analysis, comparing % overall funding relative to % overall YLL, Burnet et al. [8] addresses these issues and identifies several examples of funding discrepancies. Figure 9 presents our values of %funding/%YLL and those calculated using data in table 2 of Burnet et al. [8]. The general agreement of funding levels for different cancers (R^2^ = 0.57 for the data shown in Figure 9) based on two distinct data sets (older UK vs more recent US data) suggests that the conclusions we make from this data are likely to be robust. A number of cancers (bladder, esophageal, lung, pancreatic, stomach, and uterine) are identified as underfunded in both studies.

Various societal and cancer specific factors may account for some of the discrepancies observed.

Consideration of the data and discussions with medical professionals lead us to believe that the relatively high level of funding for breast cancer is due to the organized efforts of women’s groups and charitable organizations to raise awareness and concern about the burden caused by this cancer. Similar efforts by women's groups may account for the relatively high level of funding for cervical cancer. Given this, we are unsure why uterine cancer exhibits lower than expected rates of funding.

Funding for lung cancer is quite low given its cost, mainly due to a "blame the victim" attitude in which the personal choice to smoke is seen as the direct cause [15]. The levels of funding for liver cancer (2.6% of funding compared to 3.8% of deaths and 3.8% of YLL) and oral cancer (0.5% of funding compared to 1.6% of deaths and 1.8% of YLL) may also be influenced by this “blame the victim” prejudice (Hepatitus B infection and alcoholism contribute to liver cancer risk and chewing tobacco contributes to oral cancer risk). However, this rationale does not appear to be consistently applied. Cervical cancer is largely attributable to the HPV virus and the risk of skin cancer can be greatly reduced by the application of sunblock; in both cases, cancer patients are not typically blamed for their personal actions. In fact, the per-death research funding for cervical and skin cancers are both in the top 6 of our 21 examined cancers (Figure 1b) and cervical cancer has the highest per-mortality research spending rate, indicating a prioritization of research. Morally, if we decide that it is appropriate for cancer victims to receive blame for cancers that result as a consequence of their actions, funding for cervical and skin cancers should be reduced in lieu of increased funding for other cancers. Alternatively, if we decide that it is morally unacceptable to place blame on cancer victims for poor lifestyle choices, funding for lung, liver, and oral cancers should be increased to levels comparable to other cancers relative to their burden.

Although we examined the major source of research funding provided by government, our study did not examine the research effort conducted by commercial entities and is therefore a partial view of the overall funding landscape. The main reason we omitted research funding by private entities is because the reallocation decisions we suggest in this paper are guided by the goal of reducing societal burden; private research entities are motivated by financial concerns and the burdens analysed in this paper are therefore less relevant for their decision making processes. An interesting point arises however when considering the approach taken by private entities. Private research will tend to focus on the types of cancer with the largest potential profit and private funding will therefore be higher for more common cancers which create a larger market. Several of the cancers we identified as overfunded are just these more common types (breast, prostate, leukemia) and some of the ones we identified as underfunded are relatively rare (oral, uterine, stomach) and we expect that market forces will tend to exacerbate these discrepancies in the private sector. For this reason we feel that governmental priorities should lean on the side of favoring research for cancers that are more rare when societal burden is similar.

Another factor that may account for the degree of overfunding for certain cancers may be the existence positive feedback cycles. As progress is made on one type of cancer, successful researchers publish papers that attract new funds and inspire students to follow their footsteps. This progression leads to new discoveries and the preparation of more sophisticated proposals and studies [28]. For example, consider two hypothetical research communities where the first develops cancer treatments leading to high cure rates (e.g., 99%) and the second develops fewer treatments with lower cure rates (e.g., 40%). Clearly the potential for improvement is higher in the second community. However, the success rate of the first community may outshine the second and attract more funding. This misallocation of resources may have occurred in the case of leukemia research. Branton [15] notes that hematopoietic cancers attracted considerable attention and funding for treatment. These cancers now have among the highest survival rates. If the societal dynamic we just described is a potential cause of the non-equitable distribution of research funding, specific efforts to direct researchers away from highly studied cancers to less studied ones may be warranted.

**Figure 10:**
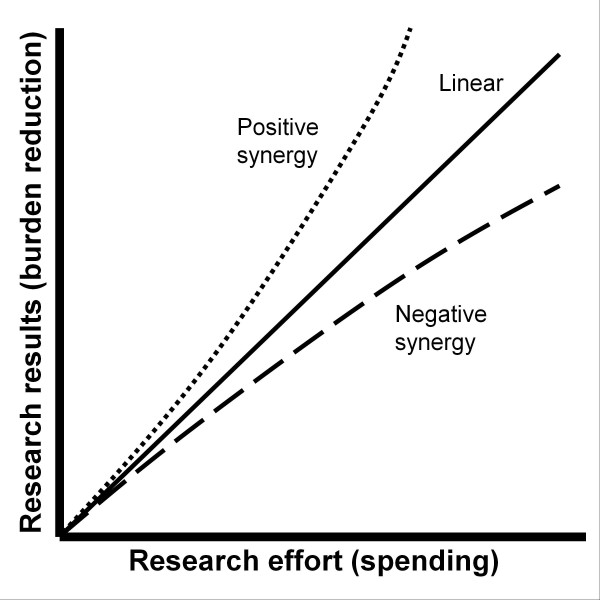
**Hypothetical relationships between research effort (I.E., dollars) and results (metric unspecified).** If each dollar spent generates results that lead to the same decrease in burden then the relationship between effort and results would be a linear one (solid line). Positive synergistic effects would cause results, per dollar, to increase with the total funding amount (dotted line). Negative synergistic effects would cause results, per dollar, to decrease with the total funding amount (dashed line). Which of these relationships is likely to be true is unclear, but each leads to different optimal approaches for the distribution of research effort.

Conversely, targeted focus on specific cancers may be beneficial depending on the nature of the relationship between research effort and results. In our study we assume a linear relationship between effort and result, but other relationships may exist (Figure 10). For example, if research efforts have a positive synergistic effect (where each additional dollar leads to a magnified result), the optimal strategy would be to concentrate funding in fewer cancers and shift the focus as each cancer gains improved treatments. Positive synergistic activities can come from increasing the diversity of research plans and funding high-risk high reward approaches that would otherwise not be tried. On the other hand, if a negative synergistic effect occurs where each additional dollar spent leads to proportionally smaller results, the optimal strategy would be to spread funding across cancers more equitably, regardless of societal burden. Negative synergistic effects can come from additional funding being allocated to projects that are similar to previous ones and offer marginal novelty or potential for benefit. In light of this, examining the relationship between funding and results to identify the type of synergistic relationship in practice may prove highly beneficial.

Currently, the NIH research funding decision process has two stages. The initial stage is the scientific review of the proposed study addressing significance, approach, and innovation. This stage generates ranking based on purely scientific factors. The second stage considers additional criteria, including public health priorities such as quality of life or health disparities. We believe studies like ours can enhance the second stage of this process by recognizing cancers that deserve more attention and improving the allocation of limited research resources.

Many may feel that reductionism to numbers in the realms of human suffering and death is cold and heartless and a number of researchers have discussed controversies associated with setting funding priorities based on measures of burden [5-7,25,26]. We strongly believe that funding decisions should take burden into account; since total funding is limited, only by objectively identifying disorders that receive non-optimal funding can we adjust our efforts to minimize the overall cost of disease. Our approach has led us to identify breast cancer, prostate cancer, and leukemia as funded at levels higher than other cancers relative to their societal burden and bladder, esophageal, liver, oral, pancreatic, stomach, and uterine cancers as relatively underfunded.

## Conclusions

We analyzed research funding distribution for different cancers in the United States. Based on burden metrics including incidences, mortalities, economic costs, and Years of Life Lost (YLL) we identified inequities in cancer research funding relative to burden. Overfunded cancers include breast cancer, prostate cancer, and leukemia; underfunded cancers include bladder, esophageal, liver, oral, pancreatic, stomach, and uterine cancer. We recommend redistribution from overfunded cancers to underfunded cancers to improve the effectiveness of cancer research funding.

## Competing interest

One author (AC) had a parent die from pancreatic cancer, the other (CN) has a parent currently suffering from breast cancer.

## Authors’ contributions

AC participated in the design of the study, performed the statistical analysis, and drafted the manuscript. CN carried out the collection of the data, participated in the literature search, and assisted in drafting the manuscript. All authors read and approved the final manuscript.

## Pre-publication history

The pre-publication history for this paper can be accessed here:

http://www.biomedcentral.com/1471-2458/12/526/prepub
